# Effect of Endocytosis Inhibitors on the Cytotoxicity and Antitumor Activity of Anti-GD2 ADCs

**DOI:** 10.32607/actanaturae.27900

**Published:** 2026

**Authors:** M. M. Titov, I. V. Kholodenko, D.V. Kalinovsky, A. O. Makarova, D. Y. Ryazantsev, E. V. Svirshchevskaya, S. M. Deyev, R. V. Kholodenko

**Affiliations:** Shemyakin–Ovchinnikov Institute of Bioorganic Chemistry, Russian Academy of Sciences, Moscow, 117997 Russia; Orekhovich Institute of Biomedical Chemistry, Moscow, 119121 Russia; National Research Ogarev Mordovia State University, Saransk, 430005 Russia; Institute of Fundamental Medicine and Biology, Kazan Federal University, Kazan, 420008 Russia; Real Target LLC, Moscow, 108841 Russia

**Keywords:** internalization, endocytosis, ganglioside GD2, monoclonal antibodies, antibody–drug conjugates, cancer immunotherapy

## Abstract

Cancer remains a critical public health challenge, and developing novel approaches to cancer therapy is highly relevant. Targeted tumor therapy using antibody–drug conjugates (ADCs) has already demonstrated its efficacy in multiple tumors. Ganglioside GD2 is a promising target for ADC development. However, its functional properties, which are important for the cytotoxicity of ADCs, remain poorly understood. This study focuses on the mechanisms of receptor-mediated endocytosis for the conjugate formed between the anti-GD2 antibody ch14.18 and monomethyl auristatin E (MMAE), as well as the approaches for modulating this process using endocytosis inhibitors. Our findings demonstrate that anti-GD2 ADCs are efficiently internalized into tumor cells through various endocytic pathways, including the clathrin- and caveolin-mediated pathways, as well as macropinocytosis. The efficiency of ADC accumulation directly correlates with the level of GD2 expression on the tumor cell surface and is determined by the properties of the parental antibody, independent of the cytotoxic payload. Endocytosis inhibitors can modulate the functional properties of anti-GD2 ADCs, either decreasing or increasing the cytotoxic effects of the conjugates in GD2-positive cells. Nystatin, a specific inhibitor of caveolin-mediated endocytosis, increased ADC accumulation in cells and reduced the IC_50_ 1.5- to 2.5-fold depending on the cell line. Although administration of nystatin together with anti-GD2 ADCs did not enhance tumor growth inhibition compared to ADC monotherapy in the GD2- positive mouse tumor model, further optimization of combination therapy conditions aimed at enhancing receptor-mediated endocytosis is a promising strategy for potentiating the antitumor effects of anti-GD2 ADCs.

## INTRODUCTION

Cancer, which annually claims the lives of millions of people, is the second leading cause of mortality worldwide, ranking only behind cardiovascular diseases [1]. Conventional treatment modalities such as radiation therapy and chemotherapy have long remained the mainstay of treatment for a broad range of malignancies [2]. However, these approaches lack adequate specificity and have a narrow therapeutic index, resulting in severe adverse effects because of the off-target drug activity on healthy tissues, thereby limiting their clinical utility [3]. Immunotherapy based on monoclonal antibodies (mAbs) has enabled targeted action against tumor cells, substantially reducing adverse effects and refocusing strategies for cancer therapy toward a more favorable safety profile [4]. Nevertheless, monoclonal antibody therapy exhibits limited efficacy in certain cases compared to conventional chemotherapy [5].

An unmet medical need – namely, the lack of effective and safe antitumor therapeutics capable of confining their cytotoxic activity to the tumor growth site while exerting reduced toxicity toward healthy tissues – has driven years of research and development, culminating in the successful implementation of the antibody–drug conjugate (ADC) strategy [6, 7]. Clinical trials and routine clinical practice have demonstrated that ADCs are superior to combination tumor therapy comprising unmodified antibodies and chemotherapeutic agents. There is evidence to suggest that ADCs will become the most sought-after targeted cancer therapeutics in the near future [8, 9].

In selecting a target molecule for an ADC under development, its internalization capacity following the antigen–antibody complex formation plays a crucial role [10]. The rate and efficiency of tumor marker endocytosis largely determine the efficacy of the ADC. For the drug to be cytotoxic, it needs to be internalized and delivered to lysosomes, where it is released from the conjugate and acts on the tumor cell. Robust endocytosis of the ADC results in significant accumulation and release of the active ingredient, thereby promoting rapid tumor cell elimination [11]. The endocytic efficiency of the ADC/tumor marker complex depends on the properties of both the target antigen and the antigen-specific antibody within the ADC. Furthermore, the drug payload per se can also affect the internalization and intracellular travel of the ADC [12, 13].

Collectively, these observations suggest a strong advantage of the strategies aiming to enhance receptor-mediated endocytosis across multiple domains of antitumor therapy, including ADC development [14]. Modulation of the molecular endocytic pathways is such a strategy.

Thus, nystatin, an inhibitor of caveolin-mediated endocytosis (CvME), has been shown to potentiate the cytotoxicity and antitumor activity of an anti-EGFR ADC both in vitro and in vivo [15]. The observed effects of nystatin are presumably attributable to the superior efficiency of the classical clathrin-mediated endocytosis (CME) compared to CvME, as well as to the fact that blockade of CvME shifts internalization toward a faster mechanism, thereby promoting greater accumulation and release of the cytotoxic payload inside a tumor cell. Nystatin-induced switching of endocytosis from CvME to CME was also found to boost the internalization and activity of endostatin in endothelial cells [16]. Chemical endocytosis inhibitors have been reported to significantly affect the internalization of lipoplexes and polyplexes [17, 18]. Inhibition of CME by chlorpromazine (CPZ) or upon potassium ion depletion resulted in the suppression of DOTAP/DNA lipoplex endocytosis in A549 and HeLa tumor cell lines, while CvME inhibitors, filipin and genistein, did not appreciably affect lipoplex accumulation in the tumor cells. All the tested inhibitors showed diminished endocytosis efficiency upon PEI/DNA polyplex internalization. These findings underscore the importance of CME for lipoplex internalization and suggest that both endocytic pathways are involved in polyplex endocytosis [17]. It has been demonstrated using SK-HEP1 human liver adenocarcinoma cells that inhibitors of CME and macropinocytosis reduce the accumulation of cholesterol-containing liposomes, whereas the CvME inhibitors nystatin and filipin III enhance the endocytosis of these liposomes into tumor cells [18].

We have demonstrated that the tumor-associated ganglioside GD2 is a promising target for developing ADCs, as it is highly expressed on the cells of various tumor types, while being minimally expressed in normal cells. Moreover, tumor cells are characterized by a high level of endocytosis of GD2 when bound into a complex with GD2-specific antibodies of various formats [19, 20]. The antibody–GD2 complex is internalized via a mixed mechanism involving CME, CvME, and macropinocytosis [21].

Our study aimed to investigate the effect of endocytosis modulation on the cytotoxicity and antitumor properties of anti-GD2 ADC both in vitro and in vivo. We compared the mechanisms of endocytosis of antiGD2 mAb and ADC into several GD2-positive tumor cell lines; we evaluated the effects of the inhibitors of CME, CvME, and macropinocytosis on the cytotoxic activity of anti-GD2 ADC in GD2-positive tumor lines; as well as assessed the influence of nystatin, a specific CvME inhibitor, in a murine model of GD2- positive cancer.

## EXPERIMENTAL

The following reagents were employed in this study: GD2-specific antibodies Ch14.18 (Dinutuximab), nystatin (Nyst), mcvcMMAE (MC-Val-Cit-PABC-MMAE), methyl-β-cyclodextrin (MβCD), chlorpromazine (CPZ), cytochalasin D (CytoD) (MedChemExpress, USA); ganglioside GD2, 3-[[4,5]-dimethylthiazol-2-yl]-2,5-diphenyltetrazolium bromide (MTT), Tris, EDTA, horseradish peroxidase (HRP)-conjugated antibodies against human immunoglobulins, tris(2-carboxyethyl)phosphine hydrochloride (TCEP), Tween-20 (Sigma-Aldrich, USA); fetal bovine serum (FBS; HyClone, USA); RPMI-1640, DMEM-F12, penicillin, streptomycin, Trypsin/Versene solution, L-glutamine, PBS (PanEco, Russia); 3,3’,5,5’-tetramethylbenzidine (TMB substrate) (1-Step Ultra TMB-ELISA Solution) (Thermo Fisher, USA); and nonfat dry milk (Roth, Germany).

**Modification of GD2-specific mAbs and ADC with a pH-sensitive dye**

The anti-GD2 ADC and anti-GD2 mAbs labeled with the pH-sensitive dye pHAb Dye (mAb-pHAb) were generated and purified according to the protocols reported earlier [19, 21].

For generating a conjugate simultaneously carrying the pH-sensitive dye and the microtubule polymerization inhibitor MMAE (anti-GD2 ADC-pHAb), a maleimide-activated MMAE toxin and pHAb Dye were added into the antibody solution at equimolar ratios (5 : 1 molar excess of both the drug and dye over the protein), and the mixture was incubated for 2 h at 37°C under agitation on a shaker. Unreacted mcvcMMAE and the pHAb Dye were removed from the conjugate on Zeba Spin Desalting Columns (7K MWCO). The resulting conjugates were sterilized by filtration through a 0.22 μm membrane. The degree of labeling (DOL) of the pHAb Dye to the antibody was determined by UV-visible spectroscopy, according to the manufacturer’s protocol (Promega, USA).

**Size exclusion chromatography (SEC)**

The purity and aggregation degree of the resulting conjugates were assessed by SEC according to the protocol described previously [22]. An analysis was conducted on an SCG-P-030-V8 chromatography system (Yocell Biotechnology, China) equipped with a Superdex 200 Increase 10/300 GL column (Cytiva, USA) or a TSK-GEL 2000SW I.D × L. 7.5 × 600 mm Spherogel column (Beckmann, USA) at flow rates of 0.5 and 1 mL/min, respectively. PBS supplemented with 15% acetonitrile was used as the mobile phase. The chromatograms were processed using the SCG software (Yocell Biotechnology, China).

**Assessment of the binding of anti-GD2 mAb-pHAb and ADC-pHAb to ganglioside GD2 by direct ELISA**

Binding was assessed using a previously reported method [23] with some modifications. Serial dilutions of anti-GD2 mAb and anti-GD2 ADC, as well as their pHAb Dye-labeled derivatives, were added into wells pre-coated with the adsorbed GD2. Once incubation had been completed, HRP-conjugated secondary antibodies (1 :  6000 dilution) were added. After further incubation and washing steps, TMB solution was added into the wells. Absorbance was measured at 450 nm using a DEL-100 microplate spectrophotometer (Miulab, China).

**Cell lines**

The following cell lines were used in this study: the B78-D14 murine melanoma cell line; the IMR-32 human neuroblastoma cell line; the T98G human glioma cell line; and the Hs578t human breast cancer cell line (collection of the Institute of Bioorganic Chemistry, Russian Academy of Sciences). The GD2- positive B78-D14 murine melanoma cell line, generated by transfecting the GD2-negative B16 line with the genes encoding the GD3 and GD2 synthases [24], was kindly provided by David Schrama (University Hospital of Würzburg, Germany).

IMR-32, T98G, and Hs578t cells were cultured in DMEM/F12 medium supplemented with 10% FBS, L-glutamine, and antibiotics at 37°C in a 5% CO_2_ incubator. The B16 and B78-D14 cell lines were cultured in RPMI-1640 medium containing identical supplements.

**Spectrofluorometric and cytofluorometric analyses of the internalization of anti-GD2 mAb-pHAb and ADC-pHAb**

A spectrofluorometric analysis of conjugate internalization by tumor cells was performed according to the procedure described previously [21], with some modifications. Cells were seeded onto 96-well plates (Greiner, Austria). After 24 h, the culture medium was replaced and serial dilutions of anti-GD2 mAb-pHAb or anti-GD2 ADC-pHAb (starting at 50 nM with subsequent twofold dilutions) were added, followed by a 24 h incubation period. After incubation and two washes with PBS, fluorescence was measured using a GloMax-Multi Detection System microplate reader (Promega) equipped with an Ex 525 nm/Em 580–640 nm filter.

To conduct a cytofluorometric analysis of internalization, cells were seeded onto 12-well plates (Greiner, Austria). After 24 h, the medium was replaced and anti-GD2 mAb-pHAb or ADC-pHAb (5 μg/mL) was added. In the experiments involving endocytosis inhibitors, the cells were pre-incubated with them for 30 min before adding the conjugate. Next, the cells were incubated for 6 or 24 h, detached from the culture plastic using Trypsin/Versene solution, and washed with PBS. Measurements for the samples were performed on a LongCyte C3080 flow cytometer (Beijing Challen Biotechnology Co., China), with a minimum of 5 × 10^3^ cells recorded per sample. Data were analyzed using the FlowJo 10.8.1 software (Becton, Dickinson and Company, USA). In order to assess the effect of endocytosis inhibitors, the degree of internalization (%) was determined by calculating the relative fluorescence intensity (RFI) of each experimental sample, defined as the ratio between the mean fluorescence intensities (MFI) of the sample and the autofluorescence control. The RFI of the sample stained with anti-GD2 mAb-pHAb (or ADC-pHAb) was taken as 100% internalization. The degree of internalization (%) in inhibitor-treated samples was calculated as (RFI I / RFI C) × 100, where I denotes cells incubated with the endocytosis inhibitor and anti-GD2 mAb-pHAb (or ADC-pHAb), and C denotes cells incubated with anti-GD2 mAb-pHAb (or ADC-pHAb) alone.

**The effect of endocytosis inhibitors on the cytotoxicity of anti-GD2 ADCs**

The effect of endocytosis inhibitors on the cytotoxicity of anti-GD2 ADCs was studied using the MTT assay following a standard colorimetric protocol [25].

The cytotoxicity of the endocytosis inhibitors toward the selected cell lines was preliminarily assessed in the MTT assay. Cells were incubated with serial dilutions of the inhibitors for 72 h. Following standard procedures, the absorbance of a formazan solution was measured at 570 nm using a DEL-100 microplate reader (Miulab, China). The IC_50_ and IC20 values for each inhibitor were determined.

To investigate the effect of endocytosis inhibitors on the cytotoxicity of the anti-GD2 ADC, cells were pre-incubated with endocytosis inhibitors at IC20 concentrations at 37°C for 30 min, followed by addition of serial dilutions of the ADC. The cells were then incubated with the specimens for 72 h, and absorbance was measured using the procedure described above. Cell viability was calculated using the formula: (OD of cells treated with anti-GD2 ADC – OD of blank wells) / (OD of control cells – OD of blank wells) × 100%, where OD of blank wells corresponds to the control wells containing no cells, with MTT and DMSO added.

For viability calculations in cells pre-incubated with endocytosis inhibitors, the absorbance of the cells incubated with the corresponding inhibitor in the absence of anti-GD2 ADC was used as the OD of the control cells.

**The effect of nystatin on the in vivo antitumor activity of anti-GD2 ADC**

The effect of nystatin on the antitumor activity of the anti-GD2 ADC was studied in a syngeneic GD2-positive B78-D14 murine melanoma model. Female C57BL/6 mice aged 6–8 weeks were used. All the experiments involving mice were approved by the Animal Care Committee of the Shemyakin– Ovchinnikov Institute of Bioorganic Chemistry RAS (Protocol No. 325, 2021) and were conducted in compliance with the AAALAC guidelines.

Subcutaneous tumors were induced by inoculating 4 × 10^6^ B78-D14 cells into the right flank of mice. The mice were randomly allocated into three groups (n = 4 per group). Ten days after the inoculation, the mean tumor size in the groups was approximately 100 mm3 . In the first and second groups, mice received three intravenous injections of 5 mg/mL anti-GD2 ADC (DAR 4) at 4-day intervals. Mice in group 2 additionally received intraperitoneal injections of 4 mg/mL nystatin according to the same schedule (three injections at 4-day intervals). Mice in group 3 (control) received PBS according to the same schedule.

Tumor volume was measured at least twice a week using the modified ellipsoid formula V = (length × width2 )/2, where length and width are the largest and second largest perpendicular linear dimensions of the tumor, respectively [26]. All the animals were euthanized when the mean tumor volume in the control group reached approximately 2,000 mm3 . The tumor growth inhibition parameter [TGI (%) = (1 – mean change in the tumor volume in the experimental group / mean change in tumor volume in the control group) × 100%] was used to determine the degree of inhibition (%) and evaluate antitumor efficacy [27].

**Statistical analysis**

Graphs were generated using SigmaPlot, MS Excel, and GraphPad Prism 8 software. Data are presented as the mean ± standard error of the mean (SEM) from at least three independent experiments or a single representative experiment out of three independent replicate experiments. Statistical analysis was performed using Student’s t-test. P-values < 0.05 were considered statistically significant.

## RESULTS AND DISCUSSION

**Preparation and analysis of anti-GD2 mAbs and anti-GD2 ADCs conjugated with the pH-dependent fluorescent dye pHAb Dye**

**Fig. 1:**
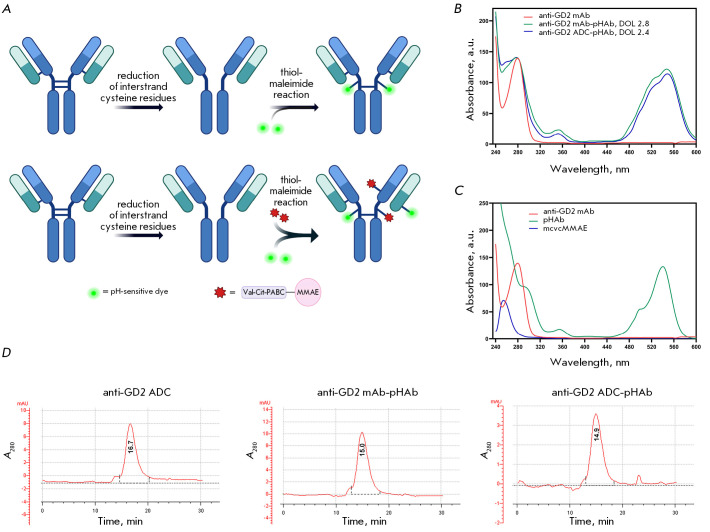
Preparation and analysis of anti-GD2 mAb-pHAb and ADC-pHAb. (A) Reaction schemes for generating anti-GD2 mAb and ADCs with the pH-sensitive dye pHAb. (B) UV-Vis analysis of the DOL in anti-GD2 mAb–pHAb and ADC–pHAb conjugates. Representative absorption spectra for anti-GD2 mAb-pHAb (molar ratio in the reaction pHAb : mAb = 5 : 1), anti-GD2 ADC-pHAb (molar ratio in the reaction pHAb : mcvcMMAE : mAb = 5 : 5 : 1), and anti-GD2 mAb, normalized at a wavelength of 280 nm. (C) Representative absorption spectra for anti-GD2 mAb, pHAb, and mcvcMMAE at the concentrations used in the conjugation reaction. (D) Size exclusion chromatography of anti-GD2 mAb, mAb-pHAb, and ADC-pHAb conjugates. The elution time of the protein is shown as a number under the peak

We have previously demonstrated that GD2-specific antibodies and their fragments exhibit a high degree of internalization by GD2-positive cells and that the pH-sensitive fluorescent dye pHAb Dye is a convenient tool for analyzing antibody endocytosis and delivery to lysosomes [21]. To compare the mechanisms and the efficiency of internalization of antibodies and the ADCs based on them, we generated anti-GD2 mAbs and anti-GD2 ADCs labeled with pHAb Dye. Full-length GD2-specific chimeric antibodies ch14.18 were used in the reaction with MMAE toxin and/or pHAb Dye. Figure 1A schematically shows the conjugate generation reaction.

The optimal protein-to-small molecule ratios were selected to achieve comparable degrees of labeling (DOL) of the pH-sensitive fluorophore to antiGD2 mAbs and anti-GD2 ADCs. The average DOL values for anti-GD2 mAb-pHAb and ADC-pHAb  were determined by UV-visible spectroscopy based on absorbance measurements at 280 and 532 nm. This approach yielded conjugates with comparable fluorophore DOLs – 2.8 and 2.4 for the anti-GD2 mAb-pHAb and anti-GD2 ADC-pHAb, respectively (Fig. 1B). The MMAE drug within anti-GD2 ADCpHAb did not contribute to the DOL assessment, since it shows negligible absorbance at the aforementioned wavelengths (Fig. 1C).

Conjugation of the pH-sensitive fluorophore to antiGD2 mAbs, as well as the simultaneous conjugation of pHAb Dye and MMAE to the antibodies, neither compromised antibody stability even at high dye-toprotein ratios nor resulted in aggregate formation or antibody fragmentation. The SEC data demonstrated that, following the standard purification procedures, the contents of aggregates and low-molecular-weight components were ≤ 5% for all the conjugates. The elution times of anti-GD2 mAb-pHAb and ADC-pHAb were shorter than those of the unmodified antibodies, which is attributable to their higher molecular weight (Fig. 1D).

**Fig. 2:**
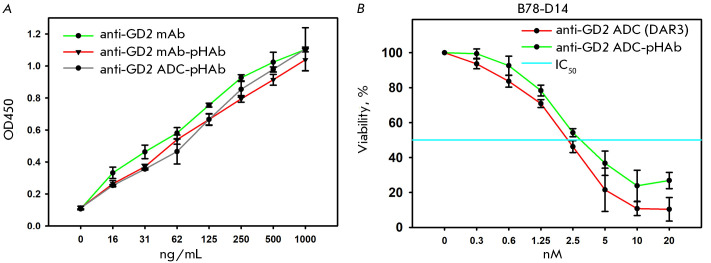
Evaluation of the antigen-binding and cytotoxic properties of the conjugates. (A) Direct ELISA, ganglioside GD2 adsorbed on the plates. Serial dilutions of anti-GD2 mAb, anti-GD2 mAb-pHAb, and anti-GD2 ADC-pHAb were added. (B) Cytotoxic activity of the anti-GD2 ADC (DAR 3) and anti-GD2 ADC-pHAb evaluated in the MTT assay. B78-D14 cells were incubated with inducers for 72 h

The binding of anti-GD2 mAb-pHAb and antiGD2 ADC-pHAb to GD2 was evaluated in comparison with the parental GD2-specific antibodies. The direct ELISA data are shown in Fig. 2B. Protein modification with maleimide-activated MMAE and/or a pHsensitive dye did not affect the ability of the antibody to interact with GD2 ganglioside, indicating that the antigen-binding capacity was preserved following the modification at thiol moieties of the protein.

An MTT assay was conducted to assess the cytotoxicity of the anti-GD2 ADC-pHAb conjugate compared to that of the corresponding anti-GD2 ADC (DAR 3) prepared using MMAE under identical conditions and at the same protein-to-toxin ratios but without the pHAb dye (Fig. 2B). Determination of the DAR for the anti-GD2-ADC-pHAb conjugate by UV-Vis spectroscopy is complicated by a spectral overlap between the MMAE toxin and the pHAb dye at 253 nm, the wavelength typically used for DAR calculation. Nevertheless, the comparable cytotoxicities of anti-GD2 ADC-pHAb (IC_50_ = 3.1 nM) and the standard anti-GD2 ADC (DAR 3) (IC_50_ = 2.2 nM) indicate that the pHAb dye does not impede MMAE conjugation and that the ADC-pHAb conjugate exhibits potent cytotoxic activity (Fig. 2B).

**Comparison of the efficiency of endocytosis of antiGD2 mAbs and anti-GD2 ADCs into tumor cells**

**Fig. 3:**
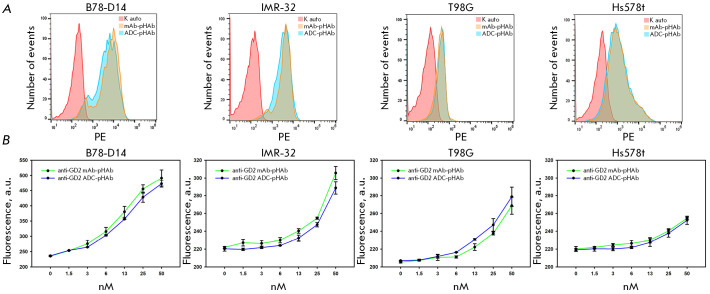
Comparative cytofluorometric (A) and spectrofluorometric (B) analysis of the internalization of anti-GD2 mAbpHAb and anti-GD2 ADC-pHAb in the B78-D14, IMR-32, T98g, and Hs578t cell lines after 24-h incubation

The DOL values of the dye were comparable in the resulting anti-GD2 mAb-pHAb and ADC-pHAb (~ three pHAb molecules per protein molecule). The efficiencies of conjugate internalization were compared by cytofluorometry and spectrofluorometry (Fig. 3).

The histograms in Fig. 3A show the cytofluorometry data. The fluorescence of the control samples (autofluorescence) is shown in red; the fluorescence of the cells following incubation with anti-GD2 mAb-pHAb and ADC-pHAb is shown in yellow and blue, respectively. Both conjugates exhibited high and comparable internalization into B78-D14 cells; the RFI values were 201 ± 25 for mAb-pHAb and 190 ± 30 for ADC-pHAb. IMR-32 cells also showed efficient internalization of both anti-GD2 mAb-pHAb (RFI 47.2 ± 7) and ADC-pHAb (RFI 49.1 ± 8). The fluorescence of the pHAb dye was weaker in T98G cells (RFI for mAb-pHAb = 3.3 ± 0.8; RFI for ADC-pHAb = 3.2 ± 1.2) and Hs578t cells (RFI for mAb-pHAb = 8.9 ± 2.6; RFI for ADC-pHAb = 9.8 ± 3.4), attesting to the reduced endocytic uptake of GD2-binding molecules into these cells. The endocytosis efficiency for both mAb-pHAb and ADC-pHAb correlated closely with the level of GD2 expression on the plasma membrane surface, which declines for the series B78-D14 > IMR-32 > T98G > Hs578t [19]. The spectrofluorometric analysis of internalization corroborated the cytofluorometry findings, revealing comparable internalization levels for mAb-pHAb and ADC-pHAb, being enhanced in B78-D14 and IMR-32 cells and less pronounced in T98G and Hs578t cells (Fig. 3B).

The most notable finding is that the degrees of internalization of anti-GD2 mAbs and ADCs are equivalent between the mAb and the ADC within each cell line, and that the efficiency of ADC accumulation directly correlates with GD2 expression levels. Therefore, ADC endocytosis is governed by the properties of the parental antibody, with no significant contribution from the conjugated drug payload.

**Comparison of the mechanisms of endocytosis of anti-GD2 mAbs and anti-GD2 ADCs into tumor cells**

The mechanisms underlying the endocytosis of anti-GD2 mAbs and anti-GD2 ADCs were investigated using the inhibitors of CME macropinocytosis (chlorpromazine (CPZ) and cytochalasin D (CytoD)), as well as CvME inhibitors (methyl-β-cyclodextrin (MβCD) and nystatin (Nyst)). CPZ disrupts the GTPase activity of dynamin, thereby predominantly inhibiting CME [28]. CytoD prevents actin polymerization, primarily blocking macropinocytosis and phagocytosis [29]. MβCD targets the lipid rafts by sequestering cholesterol from the plasma membrane, leading to the inhibition of CvME [30], while also affecting CME and macropinocytosis [31, 32]. Nyst is a sterol-binding agent that disrupts the interaction between caveolae and cholesterol in the membrane. In contrast to MβCD, nystatin is considered a selective CvME inhibitor having no effect on other endocytosis pathways [33, 34].

**Fig. 4:**
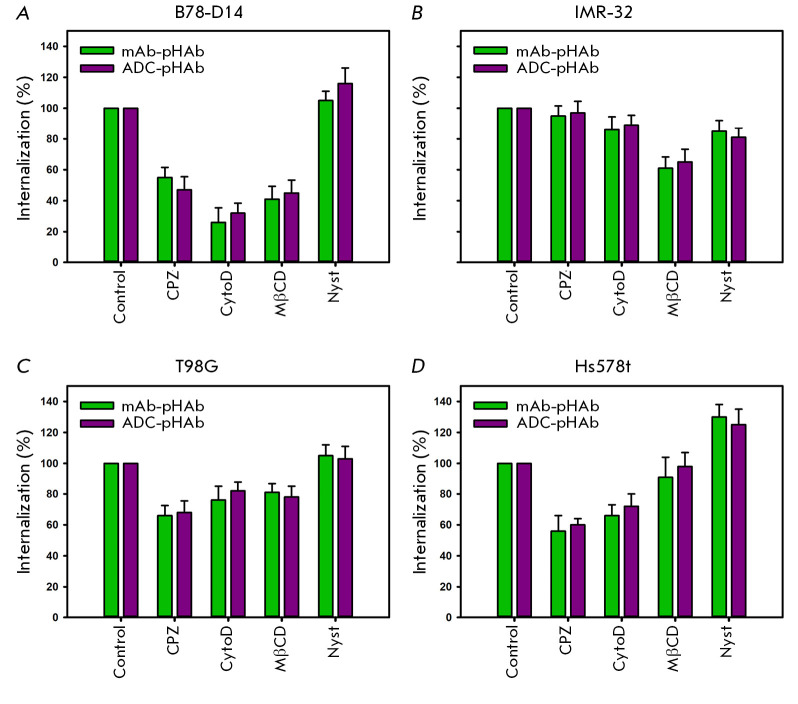
The effect of endocytosis inhibitors on the internalization efficiency of anti-GD2 mAbs and ADCs labeled with a pH-sensitive dye in B78-D14 (A), IMR-32 (B), T98g (C), and Hs578t (D) cell lines after 24-h incubation. The RFI of the control sample with anti-GD2 mAb-pHAb or ADC-pHAb added was taken as 100% internalization

B78-D14, IMR-32, T98G, and Hs578t cells were incubated with the endocytosis inhibitors at selected concentrations not exceeding their IC20 values (1 mM MβCD, 40 μM Nyst, 0.5 μM CytoD, and 7.5 μM CPZ). Anti-GD2 mAbs and ADCs labeled with the pH-sensitive dye were then added; after 24 h of incubation, the cells were analyzed by cytofluorometry. The degree of internalization and the effects of endocytosis inhibitors were assessed (Fig. 4).

The effects of endocytosis inhibitors on the internalization of anti-GD2 mAbs and ADCs were consistent across all the cell lines studied, although the endocytosis mechanisms for these molecules differed substantially depending on the cell type. The CME inhibitor CPZ suppressed the endocytosis of both mAbs and ADCs into B78-D14 cells (the degree of inhibition being 45 ± 6.5% and 53 ± 8.5%, respectively), T98G cells (the degree of inhibition being 34 ± 6.5% and 32 ± 7.4%), and Hs578t cells (the degree of inhibition being 45 ± 10% and 40 ± 4%) but exerted only a minimal effect on endocytosis into IMR-32 cells (the degree of inhibition being 5 ± 4% and 3 ± 4%). In a similar manner, the macropinocytosis inhibitor CytoD potently inhibited the internalization of both mAbs and ADCs into the B78-D14, T98G, and Hs578t cells, the degree of inhibition being as high as 70%, whereas in IMR-32 cells, the degree of inhibition was ≤ 10% (Fig. 4). These findings indicate that CME and macropinocytosis contribute significantly to anti-GD2 endocytosis in B78-D14, T98G, and Hs578t cells, while playing only a minor role in IMR-32 cells.

The CvME inhibitors MβCD and Nyst, at the concentrations tested, exerted different effects on the endocytosis of GD2-specific anti-GD2 mAbs and ADCs. Whereas MβCD inhibited internalization across all cell lines, Nyst exerted an inhibitory effect only in IMR-32 cells. Not only did Nyst fail to inhibit endocytosis in the B78-D14, T98G, and Hs578t cells, but it actually enhanced it by 15–25%, this effect being particularly pronounced in the B78-D14 and Hs578t cells (Fig. 4). These findings can be ascribed to the fact that endocytosis efficiency is pathway-dependent: when a slower pathway is switched off or inhibited, faster internalization pathways become predominant, thus increasing the intracellular accumulation of mAbs and ADCs. A number of studies have shown that CvME is a less efficient pathway [15, 16], and its selective inhibitor Nyst, unlike the broad-specificity inhibitor MβCD, redirects endocytosis toward CME and macropinocytosis, both active in the B78-D14, T98G, and Hs578t cell lines, thereby enhancing internalization. In IMR-32 cells, where CvME constitutes the primary endocytosis route, treatment with Nyst inhibits endocytosis, since the contribution of alternative pathways is negligible.

**The effect of endocytosis inhibitors on the cytotoxicity of anti-GD2 ADCs in vitro**

**Fig. 5:**
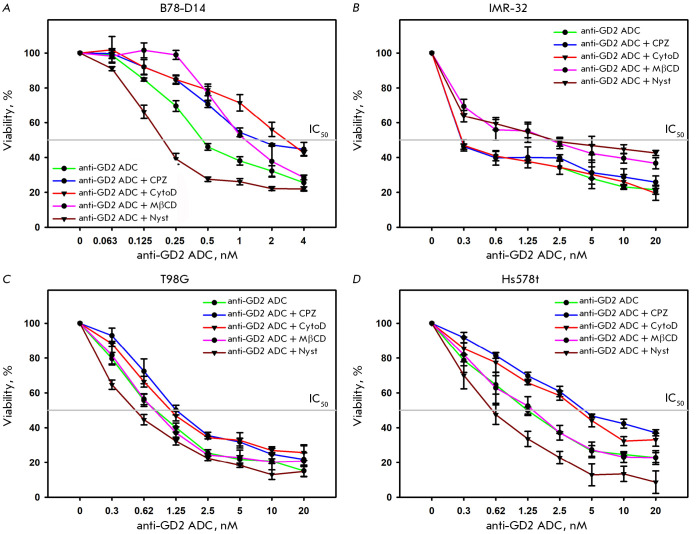
Cytotoxic effects of the anti-GD2 ADC in the B78-D14 (A), IMR-32 (B), T98g (C), and Hs578t (D) cell lines after co-incubation with the endocytosis inhibitors CPZ, CytoD, MβCD, and Nyst. The MTT assay

In order to assess the effect of endocytosis inhibitors on the cytotoxicity of ADCs comprising ch14.18 antibodies bound to the MMAE payload via a Val-Cit linker, the effects of the conjugates on the GD2-positive cell lines B78-D14, IMR-32, T98G, and Hs578t were analyzed in the MTT assay (Fig. 5). After pre-incubating cells with the selected endocytosis inhibitors, serial dilutions of anti-GD2 ADC were added.

In B78-D14 cells, the inhibitors CPZ, CytoD, and MβCD reduced the cytotoxicity of the anti-GD2 ADC, increasing the IC_50_ value 3.3-, 4.8-, and 2.2-fold, respectively. In T98G and Hs578t cells, CPZ and CytoD reduced the cytotoxicity of the ADC 2- to 3-fold, whereas MβCD had no significant effect on the IC_50_ value of the drug in these cells. Conversely, the Nyst inhibitor enhanced the cytotoxicity of the ADC in B78-D14, T98G, and Hs578t cells, reducing the IC_50_ value 2.1-, 2.3-, and 1.6-fold, respectively. No effect of CPZ or CytoD on the cytotoxicity of the ADC was observed in IMR-32 cells; however, the CvME inhibitors MβCD and Nyst significantly elevated the IC_50_ value, indicating that the efficacy of the anti-GD2 ADC is reduced in this cell line (Fig. 5).

Overall, these findings corroborate the results of the inhibition assay of the endocytosis mechanisms performed using the pHAb dye for the selected cell lines. It was demonstrated that the lower intracellular accumulation of anti-GD2 ADCs induced by treatment with endocytosis inhibitors reduced the cytotoxic activity of the drug, whereas enhanced internalization of the ADC in the presence of endocytosis inhibitors correlated with increased drug efficacy.

**The effect of the endocytosis inhibitor Nyst on the antitumor activity of the anti-GD2 ADC in vivo**

**Fig. 6:**
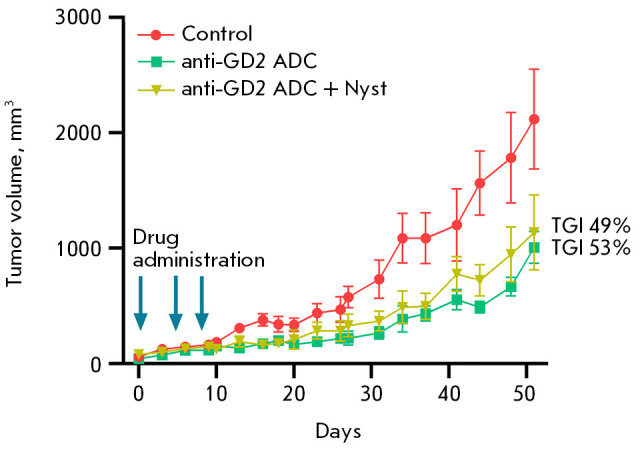
Evaluation of the effect of Nyst on the antitumor activity of the anti-GD2 ADC in the syngeneic B78-D14 melanoma model

Since nystatin, which promotes anti-GD2 ADC internalization, also potentiated the in vitro cytotoxicity of the drug in several cell lines, its effect on the antitumor efficacy of the conjugate was evaluated in a syngeneic GD2-positive murine B78-D14 melanoma model (Fig. 6).

Administration of anti-GD2 ADCs resulted in tumor growth inhibition compared to the control group (TGI 53%). In the group of mice receiving a combination of the anti-GD2 ADC and Nyst, tumor growth inhibition was also observed (TGI 49%), comparable to that in the monotherapy group (Fig. 6). Hence, although nystatin significantly potentiated the in vitro cytotoxicity of the anti-GD2 ADC, its administration to mice according to the schedule under study did not enhance the antitumor efficacy of the drug.

## CONCLUSIONS

Several therapeutics belonging to the ADC class and targeting tumor-associated markers such as HER-2, TROP2, nectin-4, FRα, TF, and EGFR have already demonstrated clinical efficacy in the treatment of solid tumors [35]. There is compelling evidence to suggest that ADCs targeting the ganglioside GD2 will also become highly sought in targeted cancer therapy [19, 36]. To develop optimal anti-GD2 ADCs, one needs not only sophisticated conjugation chemistry, but also a thorough understanding of the functional properties of the tumor target. This study describes the mechanisms underlying the endocytosis of anti-GD2 ADCs as a crucial process governing the cytotoxicity of the conjugates. Our findings demonstrate that anti-GD2 ADCs are efficiently internalized, which directly depends on the level of GD2 expression on the tumor cell surface and is primarily determined by the properties of the parental antibody, with no significant contribution from the drug payload. The mechanisms underlying the endocytosis of ADCs may vary across different tumor cell types, and endocytosis inhibitors can modulate the functional properties of anti-GD2 ADCs, in some cases substantially potentiating their cytotoxicity. Further research into the regulation and enhancement of receptor-mediated endocytosis will contribute to the development of effective therapeutic strategies for GD2-positive tumors using targeted drugs, including ADCs.

## Abbreviations

DOL: degree of labeling
mAbs: monoclonal antibodies
CME: clathrin-mediated endocytosis
CvME: caveolin-mediated endocytosis
